# Severe Anti-CV2/CRMP5, Anti-Hu, and Anti-SOX1 Antibody-Positive Paraneoplastic Neurological Syndrome Associated With Tumor Recurrence During Atezolizumab Therapy

**DOI:** 10.7759/cureus.99224

**Published:** 2025-12-14

**Authors:** Rie Tohge, Kazuki Utsumi, Katsuaki Miki, Chenyao Wang, Mitsuaki Oki, Chihiro Fujii, Takayuki Kondo

**Affiliations:** 1 Neurology, Kansai Medical University Medical Center, Moriguchi, JPN; 2 Ophthalmology, Kansai Medical University Medical Center, Moriguchi, JPN

**Keywords:** anti-cv2 antibody, anti-hu antibody, anti-sox1 antibody, atezolizumab, paraneoplastic neurological syndrome, recurrence, small cell lung cancer

## Abstract

We report a case of an 80-year-old man with small-cell lung cancer who developed paraneoplastic neurological syndrome (PNS) after four years of immune checkpoint inhibitor (ICI) therapy (atezolizumab). The patient presented with bilateral optic neuropathy, parkinsonism, and truncal ataxia. MRI revealed lesions in the basal ganglia and medial temporal lobes. Serological testing revealed a marked elevation of tumor markers, along with the presence of anti-CV2/CRMP5, anti-Hu, and anti-SOX1 antibodies. PNS onset may be primarily triggered by tumor recurrence, with long-term ICI exposure further amplifying the underlying immune response.

## Introduction

Paraneoplastic neurological syndromes (PNS) are rare autoimmune disorders occurring in association with cancer, typically mediated by immune responses to neuronal antigens aberrantly expressed by tumors [1]. PNS can affect both the central and peripheral nervous systems and exhibit a broad clinical spectrum. In addition to well-recognized presentations such as limbic encephalitis, sensory neuropathy or cerebellar ataxia, basal ganglia and optic nerve involvement may also occur, reflecting the diverse neural structures that can be targeted in paraneoplastic autoimmunity [1,2]. Small-cell lung carcinoma (SCLC) is among the malignancies most commonly associated with PNS, and multiple antibodies may coexist, although this is uncommon [2].

Immune checkpoint inhibitors (ICIs) have transformed cancer therapy by enhancing antitumor T-cell responses but can also trigger immune-related adverse events (irAEs), including neurological complications [3]. Unlike classical irAEs, which target self-antigens not expressed by tumors [4], PNS involves tumor-associated neuronal antigens [1]. Post-ICI PNS is extremely rare [5], and tumor recurrence may further influence antibody profiles and clinical presentation [6].

Here, we report a case of severe PNS in an 80-year-old man with SCLC following a four-year course of atezolizumab, presenting with simultaneous optic neuropathy, parkinsonism and truncal ataxia, and a complex antibody profile, including anti-Hu, anti-CV2/CRMP5, and anti-SOX1. This case highlights the unusual clinical context of prolonged ICI therapy, multi-antibody PNS, and recurrent SCLC, and contributes to understanding the spectrum and recognition of PNS in the era of modern immunotherapy.

## Case presentation

**Figure 1:**
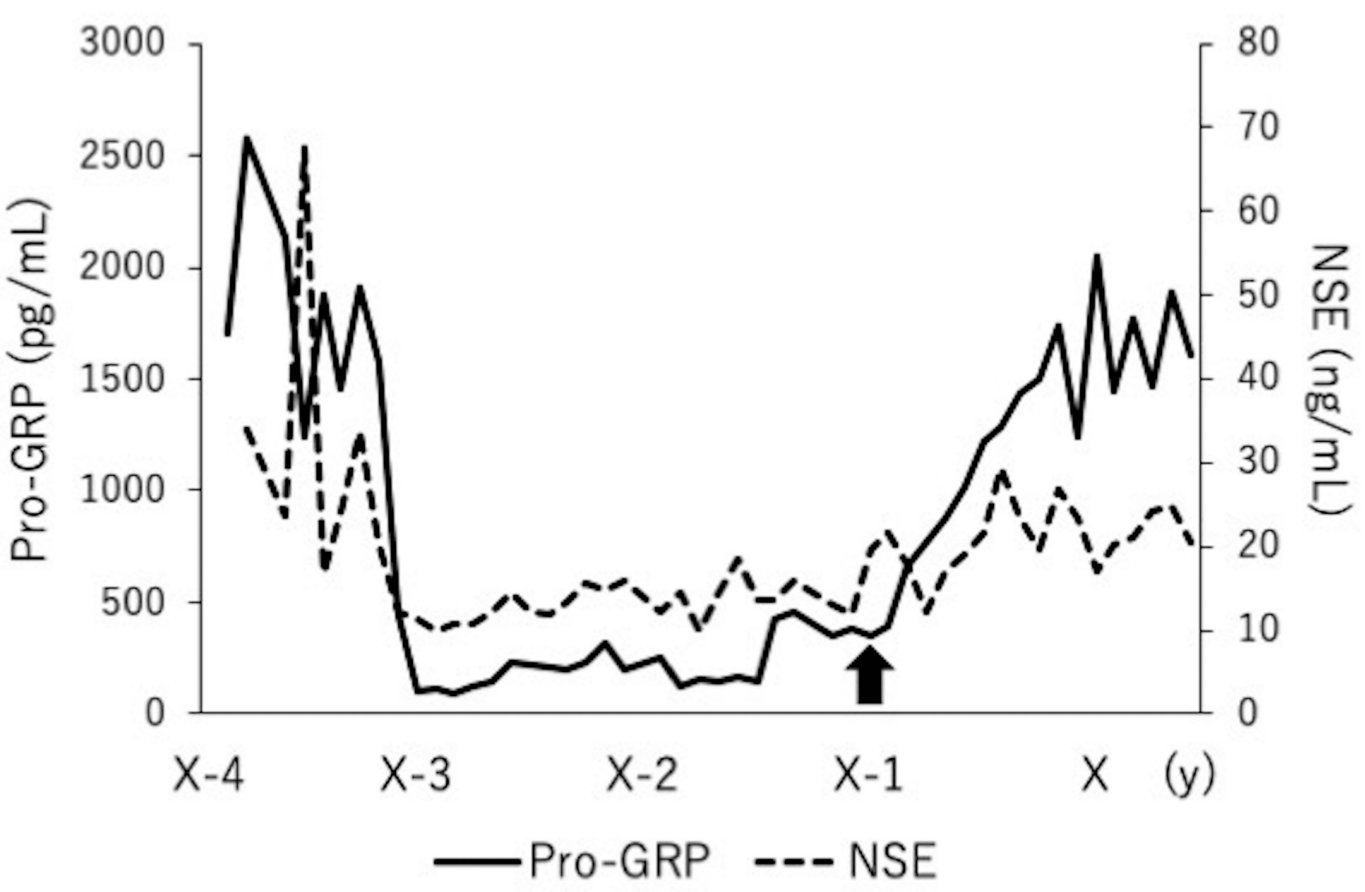
Changes in tumor markers in the patient. Pro-GRP, pro-gastrin-releasing peptide; NSE, neuron-specific enolase.

**Figure 2:**
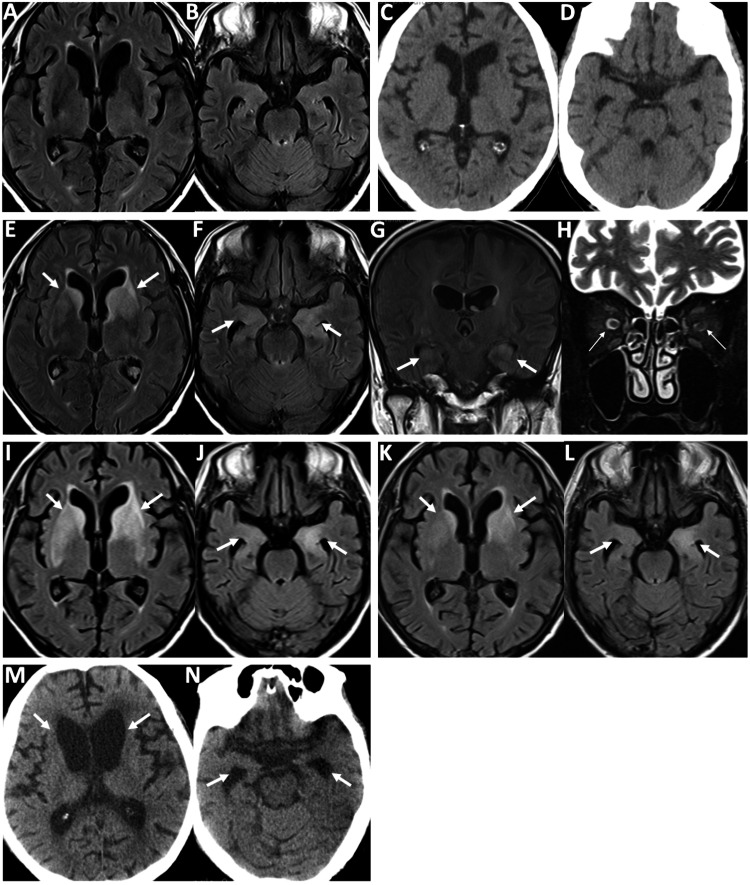
Findings from head computed tomography (CT) and brain magnetic resonance imaging during the clinical course. Brain magnetic resonance images showing no abnormal lesions after two generalized seizures (A, B). At the onset, head CT showing no space-occupying lesions or signs of increased intracranial pressure (C, D). Fluid-attenuated inversion recovery (FLAIR) imaging showing bilateral striatal and medial temporal lobe lesions, with marked left-sided predominance (E, F). Coronal FLAIR images also showed hyperintensity in the bilateral hippocampi (G). Coronal short tau inversion recovery imaging showing mild enlargement of the bilateral optic nerves without abnormal hyperintensity (H). On the sixth day of admission, FLAIR imaging revealed enlarged hyperintense lesions in the bilateral striatum (I) despite a reduction in the swollen lesions of the right medial temporal lobe (J). Subsequent FLAIR imaging showing diminished lesions in the basal ganglia and medial temporal lobes (K, L). At the final admission, head CT revealed significant atrophy of the bilateral caudate nucleus and enlargement of the anterior and inferior horns of the lateral ventricles (M, N).

An 80-year-old male patient was diagnosed with stage IVB (T4N3M1c) SCLC four years before the onset of neurological symptoms. He received four cycles of carboplatin/etoposide/atezolizumab combination therapy, followed by 52 cycles of atezolizumab monotherapy. At diagnosis, the patient’s serum tumor markers-pro-gastrin-releasing peptide (pro-GRP) and neuron-specific enolase (NSE)-were elevated at 2,580 pg/mL (normal levels: < 81 pg/mL) and 34 ng/mL (normal levels: < 15 ng/mL), respectively. As mentioned above, these levels declined after initiating chemotherapy, but began to rise again over the past year (Figure 1). Eleven months ago, he experienced two episodes of generalized seizures. The brain magnetic resonance imaging (MRI) at that time revealed no abnormal lesions (Figure 2A, 2B). Oral levetiracetam was given at a dose of 1,000 mg/day. After completing 52 cycles of atezolizumab monotherapy, the patient developed subacute, progressive bilateral visual loss and gait disturbance within four weeks. He was subsequently admitted to our hospital for a detailed evaluation. Notably, he also had a three-year history of dementia. 

**Figure 3:**
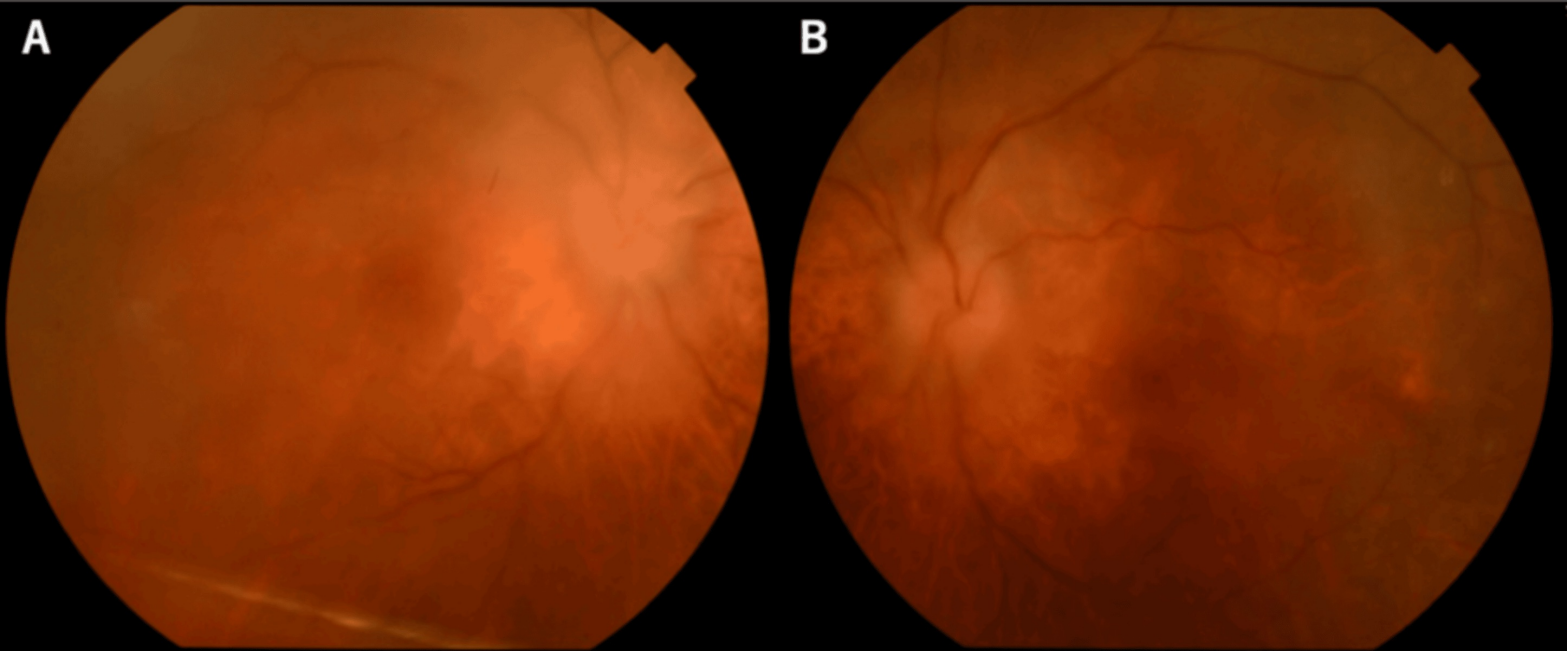
Color fundus photographs. Color fundus photographs of the right eye (A) and left eye (B) showing significant bilateral disc swelling.

Neurological examination revealed a Mini Mental Scale Examination of 19/30 [7], with no evidence of neck stiffness. Visual acuity was limited to hand motion in both eyes. The critical flicker frequency (CFF) was 23 Hz in the right eye and 22 Hz in the left eye. No abnormalities were observed in the other cranial nerves. Postural tremor and cogwheel rigidity were observed in the right upper limb. The patient was unable to stand or walk due to truncal ataxia and parkinsonism. Deep tendon reflexes in extremities were normal, and Babinski’s sign was negative in both legs. Ophthalmological evaluation revealed best-corrected visual acuity of 0.06 in the right eye and 0.08 in the left eye. Bilateral vitreous opacities and cells were observed, although no abnormalities were detected in the anterior eye segment. Fundus examination revealed marked swelling of both optic discs (Figure 3). These findings were suggestive of optic neuritis and uveitis. Head computed tomography (CT) revealed no space-occupying lesions or evidence of increased intracranial pressure (Figure 2C, 2D). Brain MRI revealed bilateral striatal and medial temporal lobe lesions on fluid-attenuated inversion recovery (FLAIR) images (Figure 2E, 2F). Coronal FLAIR imaging also demonstrated hyperintensity in the bilateral hippocampi (Figure 2G). Coronal short tau inversion recovery imaging revealed mild enlargement of the bilateral optic nerves without abnormal hyperintensity (Figure 2H). Laboratory tests revealed significantly elevated NSE levels at 20.6 ng/mL and pro-GRP at 7,073 U/L. The serum soluble interleukin-2 receptor (sIL-2R) levels were normal at 596 U/mL (normal levels: 121-613 U/mL). The patient’s antithyroid peroxidase antibody levels were mildly elevated at 7.1 IU/mL (normal levels: < 3.3 IU/mL). Meanwhile, his anti-NH2 terminal of alpha-enolase antibody was negative. Among the antineuronal antibodies associated with PNS, anti-CV2/CRMP5, anti-Hu, and anti-SOX1 antibodies were all positive (Table 1). Lumbar puncture revealed an opening pressure of 13 cm H2O, pleocytosis (24 leukocytes per mm3, 100% lymphocytes), and an elevated total protein level of 129 mg/mL. The patient’s IgG index was 1.0, and oligoclonal bands were detected. Undetectable sIL-2R was observed in the cerebrospinal fluid (CSF). CSF culture was negative, and cytological analysis revealed no malignant cells.

**Table 1:** Laboratory data on admission. NSE, neuron-specific enolase; pro-GRP, pro-gastrin-releasing peptide; sIL-2R, soluble interleukin-2 receptor; ACE, angiotensin converting enzyme; FTA-ABS, fluorescent treponemal antibody absorption test; STS, serologic test for syphilis; TP, treponema pallidum; ANA, antinuclear antibody; TPO, thyroid peroxidase; AQP4, aquaporin-4; MOG, myelin oligodendrocyte glycoprotein; NAE, anti-NH2-terminal of alpha-enolase antibody; LGI1, leucine-rich glioma-inactivated 1; CASPR2, contactin-associated protein 2; ELISA, enzyme-linked immunosorbent assay; CBA, cell-based assay; AMPH, amphiphysin; PNMA2, paraneoplastic Ma antigen 2; GAD, glutamic acid decarboxylase; DNER, delta/notch-like epidermal growth factor-related receptor.

Complete blood count	Observed value	Normal range
White blood cell (/μL)	8,100	3,500–8,500
Red blood cell (/μL)	3.5×10^6	4.0×10^6–5.7×10^6
Hemoglobin (g/dL)	11.9	12.9–17.2
Hematocrit (%)	34.7	38.2–50.8
Platelet (/μL)	13.7×10^4	14×10^4–34×10^4
Tumor biomarkers		
NSE (ng/mL)	20.6	0–15
Pro-GRP (ng/mL)	1610	0–18
sIL-2R (U/mL)	596	121–613
Biochemistry		
ACE (IU/L)	9.8	7.7–29.4
Endocrinology		
F-T4 (ng/dL)	0.93	0.9–1.7
Serum immunological test		
TFA-ABS test: STS	Negative	Negative
FTA-ABS test: TP antibody	Negative	Negative
ANA (speckled type)	1:40	<1:40
IgG (mg/dL)	2,175	870–1,700
C3 (mg/dL)	114	69–128
C4 (mg/dL)	31	14–36
Anti-SS-A/Ro antibody	Negative	Negative
Anti-thyroglobulin antibody	Negative	Negative
Anti-TPO antibody (IU/mL)	7.1	0–3.3
Anti-AQP4 antibody (ELISA/CBA)	Negative	Negative
Anti-MOG antibody (CBA)	Negative	Negative
Anti-NAE antibody	Negative	Negative
Anti-LGI1 antibody	Negative	Negative
Anti-CASPR2 antibody	Negative	Negative
Anti-neuronal antibodies		
AMPH	Negative	Negative
CV2	Positive, 2+	Negative
PNMA2	Negative	Negative
Ri	Negative	Negative
Yo	Negative	Negative
Hu	Positive, 3+	Negative
recoverin	Negative	Negative
SOX1	Positive, 2+	Negative
Titin	Negative	Negative
zic4	Negative	Negative
GAD65	Negative	Negative
Tr (DNER)	Negative	Negative

Upon admission, the patient was initially treated with intravenous methylprednisolone at 1,000 mg/day for three days, followed by oral prednisolone at 20 mg/day. However, on the sixth day of admission, the brain MRI revealed enlarged bilateral lesions in the basal ganglia (Figure 2I) despite a reduced swollen lesion in the right medial temporal lobe (Figure 2J). Therefore, the patient received add-on treatment consisting of four plasmapheresis procedures and oral azathioprine at 50 mg/day. A follow-up MRI revealed reduced lesions in the basal ganglia and medial temporal lobes (Figure 2K, 2L). Four weeks after admission, the patient regained the ability to recognize words in newspaper headlines, and his CFF values improved to 37 Hz in the right eye and 35 Hz in the left eye. His Parkinsonian symptoms in the right upper limb, as well as his gait disturbance, also improved. However, he still required assistance with standing and walking. On the 28th day of hospitalization, the patient was transferred to another facility for continued rehabilitation. 

Two months after the transfer, the patient cancelled his follow-up visit to our department due to a persistent decline in his motor and cognitive functions and was admitted to a nursing home. In addition, the patient discontinued all chemotherapy, including atezolizumab, due to a worsening performance status, despite experiencing a recurrence of SCLC in the left lower lung lobe. He ultimately died in our hospital one year after the onset of PNS due to complications of COVID-19. At the last admission, head CT revealed significant atrophy of the bilateral caudate nuclei and enlargement of the anterior and inferior horns of the lateral ventricles (Figure 2M, 2N). The autopsy was not performed. 

## Discussion

We report a case of severe PNS in an 80-year-old man with SCLC after an unusually prolonged four-year course of atezolizumab. The clinical presentation included simultaneous bilateral optic neuropathy, parkinsonism, and truncal ataxia, accompanied by MRI-confirmed lesions in the basal ganglia and medial temporal lobes. Serological testing revealed a complex autoantibody profile, including anti-CV2/CRMP5, anti-Hu, and anti-SOX1 antibodies. Based on the Graus et al. criteria, this case meets the definition of definite PNS [1].

Each of these antibodies targets intracellular neuronal antigens aberrantly expressed in SCLC [8]. CRMP5 (CV2 antigen) is normally neuronal but ectopically expressed in SCLC, inducing anti-CV2/CRMP5 antibodies [9]. Hu antigens (HuD/ELAVL4), RNA-binding proteins for neuronal differentiation, are frequently overexpressed in SCLC and key targets in anti-Hu-associated PNS [10,11]. SOX1, a neural transcription factor, is also ectopically expressed in SCLC and is considered a highly specific onconeural antigen [12].

Among the neurological symptoms, his ocular manifestations are characteristic of anti-CV2/CRMP5 antibody-associated syndromes. Unlike the isolated optic neuritis commonly observed in multiple sclerosis or neuromyelitis optica spectrum disorder, anti-CV2/CRMP5-related optic neuropathy often includes uveitis, as seen in this case [13]. 

His cerebellar ataxia may be attributable to the combined effects of all three detected antibodies-anti-CV2/CRMP5, anti-Hu, and anti-SOX1. Both anti-Hu and anti-SOX1 antibodies have been associated with cerebellar degeneration [14,15]. In contrast, CRMP5-related ataxia may reflect broader involvement of cerebellar and brainstem circuits and is frequently accompanied by additional features such as bilateral basal ganglia lesions, which can manifest clinically as parkinsonism or choreiform movements [9,16].

Limbic encephalitis, including medial temporal lobe involvement, has been associated with all three antibodies detected in this patient [17]. Notably, the response to steroid pulse therapy varied between the affected regions: while the medial temporal lobe lesions showed reduced swelling, the basal ganglia lesions progressed. This disparity in therapeutic response implies that distinct antibodies may independently drive lesion formation in different brain regions. Notably, follow-up CT performed nine months later revealed marked atrophy of the caudate nuclei. This delayed neurodegeneration is consistent with previously reported cases of anti-CV2/CRMP5 antibody-associated encephalitis [18]. However, given the known neurodegenerative potential of anti-SOX1 antibodies, their contribution to basal ganglia atrophy cannot be excluded.

In the present case, three possible mechanisms were considered for the development of PNS during long-term ICI therapy. First, tumor recurrence may have been the primary trigger, as tumor markers rose nearly a year before symptoms and recurrent SCLC was confirmed. PNS can emerge with tumor recurrence [6], and cases have been reported where PNS phenotype and anti-neuronal antibodies differed between initial diagnosis and recurrence (e.g., anti-recoverin retinopathy at diagnosis vs. anti-CV2/CRMP5 myelitis at recurrence) [19]. In this case, long-term tumor control by ICI therapy may have been followed by recurrence triggering PNS.

In addition, long-term ICI therapy may have facilitated PNS. Unlike classical irAEs targeting self-antigens [4], PNS involves immune responses to neuronal antigens ectopically expressed by tumors, suggesting distinct mechanisms [5]. Reports describe anti-Hu-positive SCLC patients with PNS worsening after ICI despite no tumor recurrence, accompanied by rising antibody titers [20]. Other studies show that anti-Hu-positive patients receiving ICIs may develop or unmask PNS, with more severe neurological deficits and higher mortality than ICI-naïve cases [11].

Finally, a synergistic mechanism is also plausible, whereby tumor relapse increases antigen burden, including sustained cytotoxic T lymphocyte (CTL) activation directly damaging neurons and the emergence of multiple autoantibodies mediating additional injury. The relative contribution of CTL-driven versus antibody-driven pathology remains uncertain.

We emphasize that the causal contribution of ICI cannot be proven in a single case, particularly given the four-year interval since initiation. The most parsimonious explanation is tumor recurrence as the primary driver, with long-term ICI exposure possibly acting as an immunological amplifier. This interpretation avoids over-attribution to ICI while recognizing the plausibility of its contribution.

Limitations include lack of peripheral nerve and spinal assessments, absence of autopsy data, and the single-case nature of this report, which prevents generalization of causal relationships between ICI, tumor recurrence, and PNS. Furthermore, the relative contributions of cytotoxic T-cell versus antibody-mediated injury remain unclear. Nonetheless, the unusual antibody profile, heterogeneous radiological responses, and context of prolonged ICI therapy make this case a valuable addition to the literature on PNS.

## Conclusions

This case illustrates the complexity of PNS arising after long-term ICI therapy, particularly in the context of tumor recurrence and multiple antineuronal antibodies. These findings underscore the need for vigilant neurological monitoring, serial antibody assessment, and further research to clarify the mechanisms linking ICI exposure, tumor dynamics, and PNS development.
